# Global maximal inequality to a class of oscillatory integrals

**DOI:** 10.1186/s13660-018-1946-x

**Published:** 2018-12-20

**Authors:** Ying Xue, Yaoming Niu

**Affiliations:** Faculty of Mathematics, Baotou Teachers’ College of Inner Mongolia University of Science and Technology, Baotou, P.R. China

**Keywords:** 42B15, 42B25, Maximal operator, Local estimate, Global estimate, Multiparameter oscillatory integrals

## Abstract

In the present paper, we give the global $L^{q}$ estimates for maximal operators generated by multiparameter oscillatory integral $S_{t,\varPhi}$, which is defined by

$$S_{t,\varPhi}f(x)=(2\pi)^{-n} \int_{\mathbb{R}^{n}} e^{ix\cdot\xi}e^{i(t_{1} \phi_{1}(|\xi_{1}|)+t_{2}\phi_{2}(|\xi_{2}|)+ \cdots+t_{n}\phi_{n}(|\xi_{n}|))}\hat{f}(\xi)\,d\xi,\quad x\in\mathbb{R}^{n}, $$

where $n\geq2$ and *f* is a Schwartz function in $\mathcal{S}(\mathbb {R}^{n})$, $t=(t_{1},t_{2},\ldots,t_{n})$, $\varPhi=(\phi_{1},\phi_{2},\ldots,\phi_{n})$, $\phi_{i}$ $(i=1,2,3,\ldots, n)$ is a function on $\mathbb {R}^{+}\rightarrow\mathbb{R}$, which has a suitable growth condition. These estimates are apparently good extensions to the results of Sjölin and Soria (J. Math. Anal. Appl 411:129–143, 2014) for the multiparameter fractional Schrödinger equation.

## Introduction and main results

Let *f* be a Schwartz function in $\mathcal{S}(\mathbb{R}^{n})$ and

$$S_{t}f(x)=u(x,t)=(2\pi)^{-n} \int_{\mathbb{R}^{n}} e^{ix\cdot\xi+it|\xi|^{a}}\hat{f}(\xi)\,d\xi,\quad (x,t)\in \mathbb{R}^{n}\times\mathbb{R}. $$

It is well known that $S_{t}f(x)$ is the solution of the fractional Schrödinger equation

1.1 $$\begin{aligned} \textstyle\begin{cases} i\partial_{t}u+(-\Delta)^{a/2} u=0,\quad (x,t)\in\mathbb {R}^{n}\times\mathbb{R},\\ u(x,0)=f(x). \end{cases}\displaystyle \end{aligned}$$

Here *f̂* denotes the Fourier transform of *f* defined by $\hat{f}(\xi)=\int_{\mathbb{R}^{n}}e^{-i\xi\cdot x}f(x)\,dx$.

We recall the homogeneous Sobolev space $\dot{H}^{s}(\mathbb{R}^{n})\ (s\in\mathbb{R})$, which is defined by

$$\dot{H}^{s}\bigl(\mathbb{R}^{n}\bigr)= \biggl\{ f\in \mathcal{S}^{\prime}: \Vert f \Vert _{H^{s}}= \biggl( \int_{\mathbb{R}^{n}} \vert \xi \vert ^{2s} \bigl\vert \hat{f}(\xi) \bigr\vert ^{2}\,d\xi \biggr)^{1/2} < \infty \biggr\} , $$

and the non-homogeneous Sobolev space $H^{s}(\mathbb{R}^{n})\ (s\in \mathbb{R})$, which is defined by

$$H^{s}\bigl(\mathbb{R}^{n}\bigr)= \biggl\{ f\in \mathcal{S}^{\prime}: \Vert f \Vert _{H^{s}}= \biggl( \int_{\mathbb{R}^{n}} \bigl(1+ \vert \xi \vert ^{2} \bigr)^{s} \bigl\vert \hat{f}(\xi) \bigr\vert ^{2}\,d\xi \biggr)^{1/2}< \infty \biggr\} . $$

Maximal operator $S^{\ast}f$ associated with the family of operators $\{S_{t}\}_{0< t<1}$ is defined by

$$S^{\ast}f(x)= \sup_{0< t< 1} \bigl\vert S_{t}f(x) \bigr\vert ,\quad x\in\mathbb{R}^{n}. $$

It is well known that if $a=2$, *u* is the solution of the Schrödinger equation

1.2 $$\begin{aligned} \textstyle\begin{cases} i\partial_{t}u-\Delta u=0,\quad (x,t)\in\mathbb{R}^{n}\times\mathbb {R},\\ u(x,0)=f(x). \end{cases}\displaystyle \end{aligned}$$

In 1979, Carleson [4] proposed a problem: if $f\in H^{s}(\mathbb{R}^{n})$ for which *s* does

1.3 $$\begin{aligned} \lim_{t\rightarrow0}u(x,t)=f(x),\quad \mbox{a.e. }x\in \mathbb{R}^{n}. \end{aligned}$$

Carleson first considered this problem for dimension $n=1$ in [4] and showed that the convergence (1.3) holds for $f\in H^{s}(\mathbb{R})$ with $s \geq\frac{1}{4}$, which is sharp was shown by Dahlberg and Kenig [8]. The higher dimensional case of convergence (1.3) has been studied by several authors, see [1, 2, 9, 11–13, 24, 25, 30, 34, 35] for example. In fact, by a standard argument, for $f\in H^{s}(\mathbb {R}^{n})$, the pointwise convergence (1.3) follows from the local estimate

1.4 $$ \bigl\Vert S^{\ast}f \bigr\Vert _{L^{q}(\mathbb {B}^{n})}\leq C \Vert f \Vert _{H^{s}(\mathbb{R}^{n})},\quad f\in H^{s}\bigl( \mathbb{R}^{n}\bigr), $$

for some $q\geq1$ and $s\in\mathbb{R}$. Here $\mathbb{B}^{n}$ is the unit ball centered at the origin in $\mathbb{R}^{n}$. On the other hand, the global estimates are of independent interest since they reveal global regularity properties of the corresponding oscillatory integrals. Next, we recall the global estimate

1.5 $$ \bigl\Vert S^{\ast}f \bigr\Vert _{L^{q}(\mathbb{R}^{n})} \leq C \Vert f \Vert _{H^{s}(\mathbb{R}^{n})}. $$

Estimate (1.5) and related questions have been well studied in literature, see, e.g., Carbery [3], Cowling [7], Kenig and Ruiz [21], Kenig, Ponce, and Vega [20], Rogers and Villarroya [29], Rogers [28], Sjölin [30–32], and so on.

For $n\geq2$ and a multiindex $a=(a_{1},a_{2},\ldots, a_{n})$, with $a_{j}>1$ and *f* being a Schwartz function in $\mathcal {S}(\mathbb{R}^{n})$, we set

$$S_{t}f(x)=(2\pi)^{-n} \int_{\mathbb{R}^{n}} e^{ix\cdot\xi}e^{i(t_{1} |\xi_{1}|^{a_{1}}+t_{2}|\xi_{2}|^{a_{2}}+ \cdots+t_{n}|\xi_{n}|^{a_{n}})}\hat{f}(\xi)\,d\xi,\quad x\in\mathbb{R}^{n}, $$

where $t=(t_{1},t_{2},\ldots, t_{n})\in\mathbb{R}^{n}$. For $n\geq2$, the local maximal operator $M^{\ast}$ is defined by

$$M^{\ast}f(x)= \sup_{0< t_{i}< 1} \bigl\vert S_{t}f(x) \bigr\vert ,\quad x\in\mathbb{R}^{n}, $$

and the global maximal operator $M^{\ast\ast}$ is defined by

$$M^{\ast\ast}f(x) = \sup_{t_{i}\in\mathbb{R}} \bigl\vert S_{t}f(x) \bigr\vert ,\quad x\in\mathbb{R}^{n}. $$

The global estimate

1.6 $$ \bigl\Vert M^{\ast\ast}f \bigr\Vert _{L^{q}(\mathbb{R}^{n})} \leq C \Vert f \Vert _{\dot{H}^{s}(\mathbb{R}^{n})} $$

and

1.7 $$ \bigl\Vert M^{\ast}f \bigr\Vert _{L^{q}(\mathbb{R}^{n})} \leq C \Vert f \Vert _{H^{s}(\mathbb{R}^{n})}. $$

In 2014, Sjolin and Soria [32] obtained the following results.

### Theorem A

([32])

*Assume* $n\geq2$. *Then*, *for every* *a*, *inequality* (1.6) *holds if and only if* $4\leq q<\infty$ *and* $s=n(\frac{1}{2}-\frac{1}{q})$.

### Theorem B

([32])

*Assume* $n\geq2$. *Then*, *for every* *a* *and for* $2< q<4$, *inequality* (1.7) *holds if and only if* $s\geq\frac {n}{2}-\frac{|a|}{4}+\frac{|a|}{q}-\frac{n}{q}$.

Multiparameter singular integrals and related operators have been well studied and raised considerable attention in harmonic analysis, which can been seen in the work of Stein and Fefferman in [14–17], and so on. In the present paper, we consider the maximal estimates associated with multiparameter oscillatory integral $S_{t,\varPhi}$ defined by

$$S_{t,\varPhi}f(x)=(2\pi)^{-n} \int_{\mathbb{R}^{n}} e^{ix\cdot\xi}e^{i(t_{1} \phi_{1}(|\xi_{1}|)+t_{2}\phi_{2}(|\xi_{2}|)+ \cdots+t_{n}\phi_{n}(|\xi_{n}|))}\hat{f}(\xi)\,d\xi,\quad x\in\mathbb{R}^{n}. $$

Here, $n\geq2$ and *f* is a Schwartz function in $\mathcal{S}(\mathbb {R}^{n})$, $\varPhi=(\phi_{1},\phi_{2},\ldots,\phi_{n})$, $\phi_{i}$ $(i=1,2,3,\ldots, n)$ is a function on $\mathbb {R}^{+}\rightarrow\mathbb{R}$. For $n\geq2$, the local maximal operator $M_{\varPhi}^{\ast}$ is defined by

$$M_{\varPhi}^{\ast}f(x)= \sup_{0< t_{i}< 1} \bigl\vert S_{t,\varPhi}f(x) \bigr\vert ,\quad x\in\mathbb{R}^{n}, $$

and the global maximal operator $M_{\varPhi}^{\ast\ast}$ is defined by

$$M_{\varPhi}^{\ast\ast}f(x)= \sup_{t_{i}\in\mathbb{R}} \bigl\vert S_{t,\varPhi}f(x) \bigr\vert ,\quad x\in\mathbb{R}^{n}. $$

The global estimates of maximal operators $M_{\varPhi}^{\ast}$ and $M_{\varPhi}^{\ast\ast}$ are defined by

1.8 $$ \bigl\Vert M_{\varPhi}^{\ast\ast}f \bigr\Vert _{L^{q}(\mathbb{R}^{n})} \leq C \Vert f \Vert _{\dot{H}^{s}(\mathbb{R}^{n})} $$

and

1.9 $$ \bigl\Vert M_{\varPhi}^{\ast}f \bigr\Vert _{L^{q}(\mathbb{R}^{n})} \leq C \Vert f \Vert _{H^{s}(\mathbb{R}^{n})}. $$

Assume that $\phi: \mathbb{R}^{+}\rightarrow\mathbb{R}$ satisfies:

1. There exists $m_{1}>1$ such that $|\phi^{\prime}(r)|\sim r^{m_{1}-1}$ and $|\phi^{\prime \prime}(r)|\gtrsim r^{m_{1}-2}$ for all $0< r<1$;
2. There exists $m_{2}>1$ such that $|\phi^{\prime}(r)|\sim r^{m_{2}-1}$ and $|\phi^{\prime \prime}(r)|\gtrsim r^{m_{2}-2}$ for all $r\geq1$;
3. Either $\phi^{\prime\prime}(r)>0$ or $\phi^{\prime \prime}(r)<0$ for all $r>0$.

Now we state our main results as follows.

### Theorem 1.1

*Assume that* $n\geq2$ *and* $\phi_{i}$ $(i=1,2,3,\ldots, n)$ *satisfies* (H1)*–*(H3). *If* $4\leq q<\infty$ *and* $s=n(\frac{1}{2}-\frac{1}{q})$, *then the global estimate* (1.8) *holds*.

### Theorem 1.2

*Let* $m=(m_{1,2},m_{2,2},\ldots,m_{n,2})$ *and set* $|m|=m_{1,2}+m_{2,2}+\cdots+m_{n,2}$. *Assume that* $n\geq2$ *and* $\phi _{i}$ $(i=1,2,3,\ldots, n)$ *satisfies* (H1)*–*(H3) *with* $m_{i,1}>1$, $m_{i,2}>1$. *Then*, *for every* *m*, *inequality* (1.9) *holds if* $2< q<4$ *and* $s\geq\frac{n}{2}-\frac{|m|}{4}+\frac{|m|}{q}-\frac{n}{q}$.

### Remark 1.1

There are many elements *ϕ* satisfying conditions (H1)–(H3), for instance, the fractional Schrödinger equation ($\phi(r)= r^{a}$), or $( \phi(r)=(1+r^{2})^{\frac{a}{2}}), (a\geq1)$, the Beam equation $(\phi(r)=\sqrt{1+r^{4}})$, the fourth-order Schrödinger equation $(\phi(r)=r^{2}+r^{4})$, iBq $(\phi(r)=r\sqrt{1+r^{2}})$, and so on (see [5, 6, 18, 19, 22, 23, 27], and the references therein). Hence, Theorem 1.1 and Theorem 1.2 imply the sufficiency part of Theorem A and Theorem B, respectively. However, due to the complexity of the symbol *ϕ*, we cannot obtain the necessities of the range of *q* in Theorem 1.1 and Theorem 1.2.

This paper is organized as follows. The proofs of Theorem 1.1 and Theorem 1.2 are given in Sect. 2 and Sect. 3, respectively. To prove Theorem 1.1 and Theorem 1.2, we next need the following important lemmas, which play a key role in proving Theorem 1.1 and Theorem 1.2, respectively. The proof of Lemma 1.4 is given in Sect. 4.

### Lemma 1.3

([26])

*Assume that* *ϕ* *satisfies* (H1)*–*(H3) *with* $m_{1}>1$, $m_{2}>1$. $\frac{1}{2}\leq s<1$ *and* $\mu\in C_{0}^{\infty}(\mathbb {R})$. *Then*

$$\biggl\vert \int_{\mathbb{R}} e^{ix\xi+it\phi( \vert \xi \vert )} \vert \xi \vert ^{-s} \mu \biggl(\frac{\xi}{N} \biggr) \,d\xi \biggr\vert \leq C \frac{1}{ \vert x \vert ^{1-s}} $$

*for* $x\in\mathbb{R}\setminus\{0\}$, $t\in\mathbb{R}$, *and* $N=1,2,3,\ldots$ . *Here the constant* *C* *may depend on* *s* *and* $m_{1}$, $m_{2}$, *and* *μ* *but not on* *x*, *t*, *or* *N*.

### Remark 1.2

The proof of Lemma 1.3 is similar to that of Lemma 2.1 in [10].

### Lemma 1.4

*Assume that* *ϕ* *satisfies* (H1)*–*(H3) *with* $m_{1}>1$, $m_{2}>1$. $\frac{1}{2}\leq\alpha\leq\frac{m_{2}}{2}$, $-1< d<1$, *and* $\mu\in C_{0}^{\infty}(\mathbb{R})$. *Then*

1.10 $$ \biggl\vert \int_{\mathbb{R}} \frac{e^{i(d\phi( \vert \xi \vert )-x\xi)}}{(1+\xi ^{2})^{\frac{\alpha}{2}}} \mu\biggl(\frac{\xi}{N} \biggr)\,d\xi \biggr\vert \leq C\frac{1}{ \vert x \vert ^{\beta}} $$

*for* $x\in\mathbb{R}\setminus\{0\}$ *and* $N=1,2,3,\ldots$ , *where* $\beta=\frac{\alpha+\frac{m_{2}}{2}-1}{m_{2}-1}$. *Here the constant* *C* *may depend on* *α* *and* $m_{1}$, $m_{2}$, *and* *μ* *but not on* *x*, *d*, *and* *N*.

### Remark 1.3

Applying the result of Lemma 1.3, the proof of Lemma 1.4 is similar to that of Lemma 2.2 in [32]. The proof of Lemma 1.4 will be given in Sect. 4.

## The proof of Theorem 1.1

Assume that $n\geq2$, $\phi_{i}$ $(i=1,2,3,\ldots ,n)$ satisfies (H1)–(H3). For $i=1,2,3,\ldots ,n$, let $t_{i}(x)$ be a measurable function on $\mathbb{R}^{n}$ with $t_{i}(x)\in\mathbb{R}$. Denote $t(x)=(t_{1}(x),t_{2}(x),\ldots,t_{n}(x))$, we set

$$\begin{aligned} S_{t(x),\varPhi}f(x)={}&(2\pi)^{-n} \int_{\mathbb{R}^{n}} e^{ix\cdot\xi}e^{i(t_{1}(x) \phi_{1}(|\xi_{1}|)+t_{2}(x)\phi_{2}(|\xi_{2}|)+ \cdots+t_{n}(x)\phi_{n}(|\xi_{n}|))}\hat{f}(\xi)\,d\xi,\\ & x\in\mathbb{R}^{n}, f\in\mathcal{S}\bigl(\mathbb{R}^{n}\bigr). \end{aligned}$$

For $4\leq q<\infty$ and $s=n(\frac{1}{2}-\frac{1}{q})$, that is, $\frac{n}{4}\leq s<\frac{n}{2}$ and $q=\frac{2n}{n-2s}$. By linearizing the maximal operator (see [30]) to prove the global estimate (1.8) holds, it suffices to show that

2.1 $$ \Vert S_{t(x),\varPhi}f \Vert _{L^{q}(\mathbb{R}^{n})}\leq C \Vert f \Vert _{\dot {H}^{s}}=C \biggl( \int_{\mathbb{R}^{n}} \vert \xi \vert ^{2s} \bigl\vert \hat{f}(\xi) \bigr\vert ^{2}\,d\xi \biggr)^{1/2}. $$

To prove (2.1) it suffices to prove that

2.2 $$ \Vert S_{t(x),\varPhi}f \Vert _{L^{q}(\mathbb{R}^{n})}\leq C \biggl( \int_{\mathbb{R}^{n}} \vert \xi_{1} \vert ^{\frac{2s}{n}} \vert \xi_{2} \vert ^{\frac{2s}{n}} |\cdots \vert \xi_{n} \vert ^{\frac{2s}{n}} \bigl\vert \hat{f}(\xi) \bigr\vert ^{2} \,d\xi \biggr)^{1/2}. $$

Let $g(\xi)=|\xi_{1}|^{\frac{s}{n}}|\xi_{2}|^{\frac{s}{n}} \cdots|\xi_{n}|^{\frac{s}{n}}\hat{f}(\xi)$, then we have

2.3 $$\begin{aligned} S_{t(x),\varPhi}f(x)={}& \int_{\mathbb{R}^{n}} e^{ix\cdot\xi}e^{i(t_{1}(x) \phi_{1}( \vert \xi_{1} \vert )+t_{2}(x)\phi_{2}( \vert \xi_{2} \vert )+ \cdots+t_{n}(x)\phi_{n}( \vert \xi_{n} \vert ))} \vert \xi_{1} \vert ^{-\frac{s}{n}} \vert \xi_{2} \vert ^{-\frac{s}{n}} \cdots \vert \xi_{n} \vert ^{-\frac{s}{n}}g(\xi)\,d \xi \\ ={}&R_{\varPhi}g(x), \end{aligned}$$

where

$$R_{\varPhi}g(x)= \int_{\mathbb{R}^{n}} e^{ix\cdot\xi}e^{i(t_{1}(x) \phi_{1}( \vert \xi_{1} \vert )+t_{2}(x)\phi_{2}( \vert \xi_{2} \vert )+ \cdots+t_{n}(x)\phi_{n}( \vert \xi_{n} \vert ))} \vert \xi_{1} \vert ^{-\frac{s}{n}} \vert \xi_{2} \vert ^{-\frac{s}{n}} \cdots \vert \xi_{n} \vert ^{-\frac{s}{n}}g(\xi)\,d \xi. $$

To prove (2.2) it suffices to prove that

2.4 $$ \Vert R_{\varPhi}g \Vert _{L^{q}(\mathbb{R}^{n})}\leq C \Vert g \Vert _{L^{2}(\mathbb{R}^{n})} $$

for *g* continuous and rapidly decreasing at infinity. We take a real-valued function $\rho\in C_{0}^{\infty}(\mathbb{R}^{n})$ such that $\rho(x)=1$ if $|x|\leq1$ and $\rho(x)=0$ if $|x|\geq2$. And we choose a real-valued function $\psi\in C_{0}^{\infty}(\mathbb{R})$ such that $\psi(x)=1$ if $|x|\leq1$ and $\psi(x)=0$ if $|x|\geq2$, and set $\sigma(\xi )=\psi(\xi_{1})\psi(\xi_{2}) \cdots\psi(\xi_{n})$. For $\xi\in\mathbb{R}^{n}$ and $N=1,2,3,\ldots$ , we set $\rho_{N}(x)=\rho(\frac{x}{N})$ and $\sigma_{N}(\xi)=\sigma(\frac{\xi}{N})$. For $x\in\mathbb{R}^{n}$, $g\in L^{2}(\mathbb{R}^{n})$, and for $N=1,2,3,\ldots$ , we define

$$\begin{aligned} R_{N,\varPhi}g(x)={}&\rho_{N}(x) \int_{\mathbb{R}^{n}} e^{ix\cdot\xi}e^{i(t_{1}(x) \phi_{1}( \vert \xi_{1} \vert )+t_{2}(x)\phi_{2}( \vert \xi_{2} \vert )+ \cdots+t_{n}(x)\phi_{n}( \vert \xi_{n} \vert ))} \vert \xi_{1} \vert ^{-\frac{s}{n}} \vert \xi_{2} \vert ^{-\frac{s}{n}} \cdots\\ &{}\times \vert \xi_{n} \vert ^{-\frac{s}{n}} \sigma_{N}(\xi)g(\xi)\,d\xi. \end{aligned}$$

The adjoint of $R_{N,\varPhi}$ is given by

$$\begin{aligned} R^{\prime}_{N,\varPhi}h(\xi)={}&\sigma_{N}(\xi) \vert \xi_{1} \vert ^{-\frac{s}{n}} \vert \xi_{2} \vert ^{-\frac{s}{n}} \cdots\\ &{}\times \vert \xi_{n} \vert ^{-\frac{s}{n}} \int_{\mathbb{R}^{2}} e^{-ix\cdot\xi}e^{-i(t_{1}(x) \phi_{1}( \vert \xi_{1} \vert )+t_{2}(x)\phi_{2}( \vert \xi_{2} \vert )+ \cdots+t_{n}(x)\phi_{n}( \vert \xi_{n} \vert ))} \rho_{N}(x)h(x)\,dx, \end{aligned}$$

where $\xi\in\mathbb{R}^{n}$ and $h\in L^{2}(\mathbb{R}^{n})$. To prove (2.4) it is sufficient to prove that

2.5 $$ \Vert R_{N,\varPhi}g \Vert _{L^{q}(\mathbb{R}^{n})}\leq C \Vert g \Vert _{L^{2}(\mathbb{R}^{n})}. $$

By duality, to prove (2.5) it suffices to show that

2.6 $$ \bigl\Vert R_{N,\varPhi}^{\prime}h \bigr\Vert _{L^{2}(\mathbb{R}^{n})}\leq C \Vert h \Vert _{L^{q^{\prime}}(\mathbb{R}^{n})}, $$

where $\frac{1}{q}+\frac{1}{q^{\prime}}=1$. Thus, we have

2.7 $$ \bigl\Vert R_{N,\varPhi}^{\prime}h \bigr\Vert _{L^{2}(\mathbb{R}^{n})}^{2} = \int \bigl\vert R^{\prime}_{N,\varPhi}h(\xi) \bigr\vert ^{2}\,d\xi = \int_{\mathbb{R}^{n}} \int_{\mathbb{R}^{n}} K_{N}(x,y)\rho_{N}(x) \rho_{N}(y)h(x)\overline{h(y)}\,dx\,dy, $$

where

2.8 $$ K_{N}(x,y)=K^{1}_{N}(x,y) K^{2}_{N}(x,y)\cdots K^{n}_{N}(x,y) $$

and

2.9 $$ K^{i}_{N}(x,y)= \int_{\mathbb{R}} \vert \xi_{i} \vert ^{-\frac{2s}{n}} e^{i(y_{i}-x_{i})\xi_{i}} e^{i(t_{i}(y)-t_{i}(x))\phi( \vert \xi_{i} \vert )} \psi_{N}(\xi_{i})^{2} \,d\xi_{i}, $$

where $i=1,2,\ldots,n$ and $N=1,2,\ldots$ . Since $\frac{n}{4}\leq s <\frac{n}{2}$, we have $\frac{1}{2}\leq \frac{2s}{n}<1$. Therefore, by Lemma 1.3, (2.9), and (2.8), we obtain

2.10 $$ \bigl\vert K_{N}(x,y) \bigr\vert \leq C \frac{1}{ \vert x_{1}-y_{1} \vert ^{1-\frac{2s}{n}}} \frac{1}{ \vert x_{2}-y_{2} \vert ^{1-\frac{2s}{n}}} \cdots\frac{1}{ \vert x_{n}-y_{n} \vert ^{1-\frac{2s}{n}}}. $$

We define

$$P_{i}f(x_{1},x_{2},\ldots, x_{n})= \int_{\mathbb{R}} \frac{1}{ \vert x_{i}-y_{i} \vert ^{1-\frac{2s}{n}}} f(x_{1}, \ldots,x_{i-1},y_{i}, x_{i+1},\ldots,x_{n}) \,dy_{i}, $$

$i=1,2,\ldots,n$. Thus, by (2.7) and (2.10), we obtain

2.11 $$\begin{aligned} &\int \bigl\vert R_{N,\varPhi}^{\prime}h(\xi) \bigr\vert ^{2}\,d\xi \\ &\quad \leq C \int_{\mathbb{R}^{n}} \int_{\mathbb{R}^{n}} \frac{1}{ \vert x_{1}-y_{1} \vert ^{1-\frac{2s}{n}}} \frac{1}{ \vert x_{2}-y_{2} \vert ^{1-\frac{2s}{n}}} \cdots \frac{1}{ \vert x_{n}-y_{n} \vert ^{1-\frac{2s}{n}}} \bigl\vert h(x) \bigr\vert \bigl\vert h(y) \bigr\vert \,dx\,dy \\ &\quad = C \int_{\mathbb{R}^{n}} \biggl( \int_{\mathbb{R}} \int_{\mathbb{R}} \int_{\mathbb{R}} \int_{\mathbb{R}}\frac{1}{ \vert x_{n}-y_{n} \vert ^{1-\frac{2s}{n}}} \frac{1}{ \vert x_{n-1}-y_{n-1} \vert ^{1-\frac{2s}{n}}}\cdots \frac {1}{ \vert x_{3}-y_{3} \vert ^{1-\frac{2s}{n}}} \\ &\qquad{}\times\frac {1}{ \vert x_{2}-y_{2} \vert ^{1-\frac{2s}{n}}} \biggl( \int\frac {1}{ \vert x_{1}-y_{1} \vert ^{1-\frac{2s}{n}}} \bigl\vert h(y_{1},y_{2}, \ldots, y_{n}) \bigr\vert \,dy_{1} \biggr) \,dy_{2}\,dy_{3}\cdots \,dy_{n} \biggr) \bigl\vert h(x) \bigr\vert \,dx \\ &\quad =C \int_{\mathbb{R}^{n}}P_{n}P_{n-1}\cdots P_{2}P_{1} \vert h \vert (x) \bigl\vert h(x) \bigr\vert \,dx. \end{aligned}$$

Invoking Hölder’s inequality, we get

2.12 $$ \int \bigl\vert R_{N,\varPhi}^{\prime}h(\xi) \bigr\vert ^{2}\,d\xi\leq C \bigl\Vert P_{n}P_{n-1}\cdots P_{2}P_{1} \vert h \vert \bigr\Vert _{L^{q}(\mathbb{R}^{n})} \Vert h \Vert _{L^{q^{\prime}}(\mathbb{R}^{n})}. $$

Since $q=\frac{2n}{n-2s}$, it follows that $q^{\prime}=\frac {2n}{n+2s}$ and the fact $\frac{1}{q}=\frac{1}{q^{\prime}}-\frac{2s}{n}$. Denote by $I_{\sigma}$ the Riesz potential of order *σ*, which is defined by

$$I_{\sigma}(f) (u)= \int_{\mathbb{R}}\frac{f(v)}{ \vert u-v \vert ^{1-\sigma}}\,dv. $$

Applying the fact $I_{s}$ is bounded from $L^{q^{\prime}}(\mathbb{R})$ to $L^{q}(\mathbb{R})$, we have

2.13 $$ \biggl( \int_{\mathbb{R}} \bigl\vert P_{j}h(x) \bigr\vert ^{q}\,dx_{j} \biggr) ^{1/q}\leq C \biggl( \int_{\mathbb{R}} \bigl\vert h(x) \bigr\vert ^{q^{\prime}} \,dx_{j} \biggr) ^{1/q^{\prime}}, $$

where $j=1,2,\ldots,n$. By (2.13) and Minkowski’s inequality, we have

2.14 $$ \bigl\Vert P_{n}P_{n-1}\cdots P_{2}P_{1} \vert h \vert \bigr\Vert _{L^{q}(\mathbb{R}^{n})} \leq C \Vert h \Vert _{L^{q^{\prime}}(\mathbb{R}^{n})}. $$

Therefore, (2.6) follows from (2.12) and (2.14). Now we complete the proof of Theorem 1.1.

## The proof of Theorem 1.2

Assume that $n\geq2$, $\phi_{i}$ $(i=1,2,3,\ldots, n)$ satisfies (H1)–(H3) with $m_{i,1}>1$, $m_{i,2}>1$. For every $m=(m_{1,2},m_{2,2},\ldots,m_{n,2})$ and $2< q<4$, we will prove that inequality (1.9) holds if $s=\frac{n}{2}-\frac{|m|}{4}+\frac{|m|}{q}-\frac{n}{q}$, where $|m|=m_{1,2}+m_{2,2}+\cdots+m_{n,2}$. For $i=1,2,3,\ldots, n$, let $t_{i}(x)$ be a measurable function on $\mathbb{R}^{n}$ with $0< t_{i}(x)<1$. Denote $t(x)=(t_{1}(x),t_{2}(x),\ldots,t_{n}(x))$, we set

$$S_{t(x),\varPhi}f(x)= \int_{\mathbb{R}^{n}} e^{ix\cdot\xi}e^{i(t_{1}(x) \phi_{1}(|\xi_{1}|)+t_{2}(x)\phi_{2}(|\xi_{2}|)+ \cdots+t_{n}(x)\phi_{n}(|\xi_{n}|))}\hat{f}(\xi)\,d\xi,\quad x\in\mathbb{R}^{n}, f\in\mathcal{S}\bigl(\mathbb{R}^{n}\bigr). $$

By linearizing the maximal operator, to prove the global estimate (1.9) it suffices to show that

3.1 $$ \Vert S_{t(x),\varPhi}f \Vert _{L^{q}(\mathbb{R}^{n})}\leq C \Vert f \Vert _{H^{s}}=C \biggl( \int_{\mathbb{R}^{n}} \bigl(1+ \vert \xi \vert ^{2} \bigr)^{s} \bigl\vert \hat{f}(\xi) \bigr\vert ^{2}\,d\xi \biggr)^{1/2}. $$

Since $s=\frac{n}{2}-\frac{|m|}{4}+\frac{|m|}{q}-\frac{n}{q} =n\frac{1}{2}-\frac{m_{1,2}+m_{2,2}+\cdots+m_{n,2}}{4} +\frac{m_{1,2}+m_{2,2}+\cdots+m_{n,2}}{q}-n\frac{1}{q} =:s_{1}+s_{2}+\cdots+s_{n}$, where $s_{i}=\frac{1}{2}-\frac{m_{i,2}}{4}+ \frac{m_{i,2}}{q}-\frac{1}{q}$, $i=1,2,\ldots,n$. Therefore, to prove (3.1) it suffices to prove that

3.2 $$ \Vert S_{t(x),\varPhi}f \Vert _{L^{q}(\mathbb{R}^{n})}\leq C \biggl( \int_{\mathbb{R}^{2}} \bigl(1+ \vert \xi_{1} \vert ^{2}\bigr)^{s_{1}}\bigl(1+ \vert \xi_{2} \vert ^{2}\bigr)^{s_{2}} \vert \cdots \vert \bigl(1+ \vert \xi_{n} \vert ^{2}\bigr)^{s_{n}} \bigl\vert \hat{f}(\xi) \bigr\vert ^{2} \,d\xi \biggr)^{1/2}. $$

Let $g(\xi)=(1+|\xi_{1}|^{2})^{\frac{s_{1}}{2}}(1+|\xi_{2}|^{2}) ^{\frac{s_{2}}{2}} \cdots(1+|\xi_{n}|^{2})^{\frac{s_{n}}{2}}\hat{f}(\xi)$, then we have

3.3 $$ S_{t(x),\varPhi}f(x)=R_{\varPhi}g(x), $$

where

$$\begin{aligned} R_{\varPhi}g(x)={}& \int_{\mathbb{R}^{n}} e^{ix\cdot\xi}e^{i(t_{1}(x) \phi_{1}( \vert \xi_{1} \vert )+t_{2}(x)\phi_{2}( \vert \xi_{2} \vert )+ \cdots+t_{n}(x)\phi_{n}( \vert \xi_{n} \vert ))} \bigl(1+ \vert \xi_{1} \vert ^{2}\bigr)^{-\frac{s_{1}}{2}}\bigl(1+ \vert \xi_{2} \vert ^{2}\bigr) ^{-\frac{s_{2}}{2}} \cdots\\ &{}\times\bigl(1+ \vert \xi_{n} \vert ^{2}\bigr)^{-\frac{s_{n}}{2}} g(\xi)\,d \xi. \end{aligned}$$

By (3.3), to prove (3.2) it is sufficient to show that

3.4 $$ \Vert R_{\varPhi}g \Vert _{L^{q}(\mathbb{R}^{n})}\leq C \Vert g \Vert _{L^{2}(\mathbb{R}^{n})} $$

for *g* continuous and rapidly decreasing at infinity. We take a real-valued function $\rho\in C_{0}^{\infty}(\mathbb{R}^{n})$ such that $\rho(x)=1$ if $|x|\leq1$ and $\rho(x)=0$ if $|x|\geq2$. And we choose a real-valued function $\psi\in C_{0}^{\infty}(\mathbb{R})$ such that $\psi(x)=1$ if $|x|\leq1$ and $\psi(x)=0$ if $|x|\geq2$, and set $\sigma(\xi )=\psi(\xi_{1})\psi(\xi_{2})\cdots\psi(\xi_{n})$ for $\xi\in \mathbb{R}^{n}$. For $N=1,2,3,\ldots$ , we set $\rho_{N}(x)=\rho (\frac{x}{N})$ and $\sigma_{N}(\xi)=\sigma(\frac{\xi}{N})$. For $x\in\mathbb{R}^{n}$, $g\in L^{2}(\mathbb{R}^{n})$, and $N=1,2,3,\ldots$ , we define

$$\begin{aligned} R_{N,\varPhi}g(x)={}& \rho_{N}(x) \int_{\mathbb{R}^{n}} e^{ix\cdot\xi}e^{i(t_{1}(x) \phi_{1}( \vert \xi_{1} \vert )+t_{2}(x)\phi_{2}( \vert \xi_{2} \vert )+ \cdots+t_{n}(x)\phi_{n}( \vert \xi_{n} \vert ))} \bigl(1+ \vert \xi_{1} \vert ^{2}\bigr)^{-\frac{s_{1}}{2}}\bigl(1+ \vert \xi_{2} \vert ^{2}\bigr) ^{-\frac{s_{2}}{2}} \\ &{}\times \cdots\times\bigl(1+ \vert \xi_{n} \vert ^{2} \bigr)^{-\frac{s_{n}}{2}} \sigma_{N}(\xi)g(\xi)\,d\xi. \end{aligned}$$

The adjoint of $R_{N,\varPhi}$ is given by

$$\begin{aligned} R^{\prime}_{N,\varPhi}h(\xi)={}& \sigma_{N}(\xi) \bigl(1+ \vert \xi_{1} \vert ^{2}\bigr)^{-\frac{s_{1}}{2}} \bigl(1+ \vert \xi_{2} \vert ^{2}\bigr) ^{-\frac{s_{2}}{2}} \cdots\bigl(1+ \vert \xi_{n} \vert ^{2}\bigr)^{-\frac{s_{n}}{2}} \int_{\mathbb{R}^{2}} e^{-ix\cdot\xi}e^{-it_{1}(x) \phi_{1}( \vert \xi_{1} \vert )} \\ &{}\times e^{-i(t_{2}(x)\phi_{2}( \vert \xi_{2} \vert )+ \cdots+t_{n}(x)\phi_{n}( \vert \xi_{n} \vert ))}\rho_{N}(x)h(x)\,dx, \end{aligned}$$

where $\xi\in\mathbb{R}^{n}$ and $h\in L^{2}(\mathbb{R}^{n})$. To prove (3.4) it suffices to prove that

3.5 $$ \Vert R_{N,\varPhi}g \Vert _{L^{q}(\mathbb{R}^{n})}\leq C \Vert g \Vert _{L^{2}(\mathbb{R}^{n})}. $$

By duality, to prove (3.5) it is sufficient to show that

3.6 $$ \bigl\Vert R_{N,\varOmega}^{\prime}h \bigr\Vert _{L^{2}(\mathbb{R}^{n})}\leq C \Vert h \Vert _{L^{q^{\prime}}(\mathbb{R}^{n})}, $$

where $\frac{1}{q}+\frac{1}{q^{\prime}}=1$. Thus, we have

3.7 $$ \bigl\Vert R_{N,\varPhi}^{\prime}h \bigr\Vert _{L^{2}(\mathbb{R}^{n})}^{2}= \int \bigl\vert R^{\prime}_{N,\varPhi}h(\xi) \bigr\vert ^{2}\,d\xi = \int_{\mathbb{R}^{n}} \int_{\mathbb{R}^{n}} K_{N}(x,y)\rho_{N}(x) \rho_{N}(y)h(x)\overline{h(y)}\,dx\,dy, $$

where

3.8 $$ K_{N}(x,y)=K^{1}_{N}(x,y) K^{2}_{N}(x,y)\cdots K^{n}_{N}(x,y), $$

and

3.9 $$ K^{i}_{N}(x,y)= \int_{\mathbb{R}}\bigl(1+\xi_{i}^{2} \bigr)^{-s_{i}} e^{i(y_{i}-x_{i})\xi_{i}} e^{i(t_{i}(y)-t_{i}(x))\phi(|\xi_{i}|)} \psi_{N}( \xi_{i})^{2}\,d\xi_{i}, $$

where $i=1,2,\ldots,n$ and $N=1,2,\ldots$ . Denote $\alpha_{i}=2s_{i}$, since $s_{i}=\frac{1}{2}-\frac{m_{i,2}}{4}+ \frac{m_{i,2}}{q}-\frac{1}{q}$, $i=1,2,\ldots,n$, and $2< q<4$, it follows that $\frac{1}{2}<\alpha_{i}<\frac{m_{i,2}}{2}$, $i=1,2,\ldots,n$. Therefore, by (3.9) and Lemma 1.4, we obtain

3.10 $$ \bigl\vert K^{i}_{N}(x,y) \bigr\vert \leq C\frac{1}{ \vert x_{i}-y_{i} \vert ^{\beta_{i}}}, $$

where $\beta_{i}=\frac{\alpha_{i}+\frac{m_{i,2}}{2}-1}{ m_{i,2}-1}$. Denote $\sigma_{i}=1-\beta_{i}$, we define

$$P_{i}f(x_{1},x_{2},\ldots, x_{n})= \int_{\mathbb{R}} \frac{1}{ \vert x_{i}-y_{i} \vert ^{1-\sigma_{i}}} f(x_{1}, \ldots,x_{i-1},y_{i}, x_{i+1},\ldots,x_{n}) \,dy_{i}, $$

$i=1,2,\ldots,n$. Thus, by (3.7), (3.8), and (3.10), we obtain

3.11 $$\begin{aligned} &\int \bigl\vert R_{N,\varPhi}^{\prime}h(\xi) \bigr\vert ^{2}\,d\xi \\ &\quad \leq C \int_{\mathbb{R}^{n}} \int_{\mathbb{R}^{n}} \frac{1}{ \vert x_{1}-y_{1} \vert ^{1-\sigma_{1}}} \frac{1}{ \vert x_{2}-y_{2} \vert ^{1-\sigma_{2}}} \cdots \frac{1}{ \vert x_{n}-y_{n} \vert ^{1-\sigma_{n}}} \bigl\vert h(x) \bigr\vert \bigl\vert h(y) \bigr\vert \,dx\,dy \\ &\quad = C \int_{\mathbb{R}^{n}} \biggl( \int_{\mathbb{R}} \int_{\mathbb{R}} \int_{\mathbb{R}} \int_{\mathbb{R}}\frac{1}{ \vert x_{n}-y_{n} \vert ^{1-\sigma_{n}}} \frac{1}{ \vert x_{n-1}-y_{n-1} \vert ^{1-\sigma_{n-1}}}\cdots \frac {1}{ \vert x_{3}-y_{3} \vert ^{1-\sigma_{3}}} \\ &\qquad{}\times\frac {1}{ \vert x_{2}-y_{2} \vert ^{1-\sigma_{2}}} \biggl( \int\frac {1}{ \vert x_{1}-y_{1} \vert ^{1-\sigma_{1}}} \bigl\vert h(y_{1},y_{2}, \ldots, y_{n}) \bigr\vert \,dy_{1} \biggr) \,dy_{2}\,dy_{3}\cdots \,dy_{n} \biggr) \bigl\vert h(x) \bigr\vert \,dx \\ &\quad = C \int_{\mathbb{R}^{n}}P_{n}P_{n-1}\cdots P_{2}P_{1} \vert h \vert (x) \bigl\vert h(x) \bigr\vert \,dx. \end{aligned}$$

Invoking Hölder’s inequality, we get

3.12 $$ \int \bigl\vert R_{N,\varPhi}^{\prime}h(\xi) \bigr\vert ^{2}\,d\xi\leq C \bigl\Vert P_{n}P_{n-1}\cdots P_{2}P_{1} \vert h \vert \bigr\Vert _{L^{q}(\mathbb{R}^{n})} \Vert h \Vert _{L^{q^{\prime}}(\mathbb{R}^{n})}. $$

Since $\beta_{i}=\frac{\alpha_{i}+\frac{m_{i,2}}{2}-1}{m_{i,2}-1}$ and $\alpha_{i}=2s_{i}$, $s_{i}=\frac{1}{2}-\frac{m_{i,2}}{4}+ \frac{m_{i,2}}{q}-\frac{1}{q}$, $i=1,2,\ldots,n$. It follows that $\beta_{i}=\frac{2}{q}$ and $\sigma_{i}=1-\beta_{i}=1-\frac {2}{q}$, $\frac{1}{q}=\frac{1}{q^{\prime}}-\sigma_{i}$. Thus, estimate (3.6) follows from (3.12) and estimate (2.14) in the proof of Theorem 1.1. Now we complete the proof of Theorem 1.2.

## The proof of Lemma 1.4

To prove Lemma 1.4, we need to present the following lemma.

### Lemma 4.1

(see [33], pp. 309–312)

*Assume that* $a< b$ *and set* $I=[a,b]$. *Let* $F\in C^{\infty}(I)$ *be real*-*valued and assume that* $\psi\in C^{\infty}(I)$.

(i) *Assume that* $|F^{\prime}(x)|\geq\lambda>0$ *for* $x\in I$ *and that* $F^{\prime}$ *is monotonic on* *I*. *Then*
  $$\biggl\vert \int_{a}^{b}e^{iF(x)}\psi(x)\,dx \biggr\vert \leq C\frac{1}{\lambda }\biggl\{ \bigl\vert \psi(b) \bigr\vert + \int_{a}^{b} \bigl\vert \psi^{\prime}(x) \bigr\vert \,dx\biggr\} , $$
  *where C does not depend on* *F*, *ψ*, *or* *I*.
(ii) *Assume that* $|F^{\prime\prime}(x)|\geq\lambda>0$ *for* $x\in I$. *Then*
  $$\biggl\vert \int_{a}^{b}e^{iF(x)}\psi(x)\,dx \biggr\vert \leq C\frac{1}{\lambda ^{1/2}}\biggl\{ \bigl\vert \psi(b) \bigr\vert + \int_{a}^{b} \bigl\vert \psi^{\prime}(x) \bigr\vert \,dx\biggr\} , $$
  *where C does not depend on* *F*, *ψ*, *or* *I*.

### Proof of Lemma 1.4

By conditions (H1) and (H2), there exist positive constants $C_{i}$ ($i=1,2,\ldots,6$) so that for $r\geq1$ and $m_{2}>1$ such that

4.1 $$ C_{1}r^{m_{2}-1}\leq \bigl\vert \phi^{\prime}(r) \bigr\vert \leq C_{2}r^{m_{2}-1} \quad\mbox{and}\quad \bigl\vert \phi^{\prime\prime}(r) \bigr\vert \geq C_{3}r^{m_{2}-2}, $$

and for $0< r<1$ and $m_{1}>1$ such that

4.2 $$ C_{4}r^{m_{1}-1}\leq \bigl\vert \phi^{\prime}(r) \bigr\vert \leq C_{5}r^{m_{1}-1} \quad\mbox{and}\quad \bigl\vert \phi^{\prime\prime}(r) \bigr\vert \geq C_{6}r^{m_{1}-2}. $$

Set

$$J= \int_{\mathbb{R}} \frac{e^{i(d\phi(|\xi|)-x\xi)}}{(1+\xi ^{2})^{\frac{\alpha}{2}}} \mu\biggl(\frac{\xi}{N} \biggr)\,d\xi. $$

To prove Lemma 1.4, it suffices to show that there exists a constant *C* such that for $x\in\mathbb{R}\setminus\{ 0\}$, $\beta=\frac{\alpha+\frac{m_{2}}{2}-1}{m_{2}-1}$ and $N\in \Bbb {N}$,

4.3 $$ \vert J \vert \leq C\frac{1}{ \vert x \vert ^{\beta}}, $$

where *C* depends only on *α*, $m_{1}$, $m_{2}$, $C_{i}$ $(i=1,2,\ldots,6)$, and *μ*.

Without loss of generality, we may assume $\xi, d>0$. Denote $\psi(\xi)={(1+\xi^{2})^{-\frac{\alpha}{2}}} \mu(\frac{\xi}{N})$, then we have

4.4 $$ \max_{\xi\geq0} \bigl\vert \psi(\xi) \bigr\vert + \int_{ 0}^{\infty} \bigl\vert \psi ^{\prime}( \xi) \bigr\vert \,d\xi\leq C. $$

In fact, since $\mu\in C_{0}^{\infty}(\mathbb{R})$ and $\frac {1}{2}\leq\alpha\leq\frac{m_{2}}{2}$, we get

4.5 $$ \max_{\xi\geq0} \bigl\vert \psi(\xi) \bigr\vert \leq C. $$

Noting that

4.6 $$ \psi^{\prime}(\xi) =-\alpha\xi{\bigl(1+\xi^{2} \bigr)^{-\frac{\alpha}{2}-1}} \mu \biggl(\frac{\xi}{N} \biggr) +{\bigl(1+ \xi^{2}\bigr)^{-\frac{\alpha}{2}}}\frac{1}{N} \mu^{\prime} \biggl(\frac{\xi}{N} \biggr), $$

we have

4.7 $$\begin{aligned} \int_{0}^{\infty} \bigl\vert \psi^{\prime}(\xi) \bigr\vert \,d\xi\leq{}&\alpha \int_{0}^{\infty}\xi{\bigl(1+\xi^{2} \bigr)^{-\frac{\alpha}{2}-1}} \biggl\vert \mu \biggl(\frac{\xi}{N} \biggr) \biggr\vert \,d\xi \\ &{}+ \int_{0}^{\infty}{\bigl(1+\xi^{2} \bigr)^{-\frac{\alpha}{2}}}\frac{1}{N} \biggl\vert \mu^{\prime} \biggl( \frac{\xi}{N} \biggr) \biggr\vert \,d\xi \\ =:{}&G_{1}+G_{2}. \end{aligned}$$

Since $\mu\in C_{0}^{\infty}(\mathbb{R})$ and $\frac{1}{2}\leq \alpha\leq\frac{m_{2}}{2}$, we obtain

4.8 $$ G_{1}\leq C \int_{0}^{\infty}\xi{\bigl(1+\xi^{2} \bigr)^{-\frac{\alpha }{2}-1}}\,d\xi =C \int_{0}^{\infty}{\bigl(1+\xi^{2} \bigr)^{-\frac{\alpha}{2}-1}}\,d\bigl(1+\xi ^{2}\bigr) =C $$

and

4.9 $$ G_{2}\leq C \int_{0}^{\infty}\frac{1}{N} \biggl\vert \mu^{\prime} \biggl(\frac{\xi}{N} \biggr) \biggr\vert \,d\xi\leq C. $$

By (4.7), (4.8), and (4.9), we get

4.10 $$ \int_{ 0}^{\infty} \bigl\vert \psi^{\prime}(\xi) \bigr\vert \,d\xi\leq C. $$

Therefore, (4.4) follows from (4.5) and (4.10).

To estimate (4.3), we choose a positive constant *M* such that $M=\max\{(\frac{1}{\delta})^{m_{2}-1},2C_{5},2\}$, where *δ* is a small positive constant such that $\delta ^{m_{2}-1}C_{2}\leq\frac{1}{2}$. Below, we show (4.3) by dividing two cases $|x|\geq M$ and $|x|< M$.

*Case* (I): $|x|\geq M$. Let $F(\xi)=d\phi(\xi)-x\xi$, we have

$$F^{\prime}(\xi)=d\phi^{\prime}(\xi)-x,\qquad F^{\prime\prime}(\xi )=d \phi^{\prime\prime}(\xi). $$

Denote $\rho=(\frac{|x|}{d})^{\frac{1}{m_{2}-1}}$, then we have $\delta\rho\geq1$. In fact, noting that $|x|\geq(\frac{1}{\delta})^{m_{2}-1}$, $0< d<1$, $m_{2}>1$, and $\frac{|x|}{d}>|x|$, it follows that $\delta\rho >\delta|x|^{\frac{1}{m_{2}-1}}\geq1$. We choose a large positive constant *λ* such that $\lambda\geq\max\{(\frac{2}{C_{1}}) ^{\frac{1}{m_{2}-1}},\delta\}$. Denote

$$I_{1}=[0,\delta\rho],\qquad I_{2}=[\delta\rho,\lambda\rho],\qquad I_{3}=[\lambda\rho,\infty). $$

Thus, we obtain

4.11 $$\begin{aligned} \vert J \vert &= \biggl\vert \int_{0}^{\infty}e^{iF(\xi)}\psi(\xi)\,d\xi \biggr\vert \leq \sum_{j=1}^{3} \biggl\vert \int_{I_{j}}e^{iF(\xi)}\psi(\xi)\,d\xi \biggr\vert =:\sum _{j=1}^{3} J_{j}. \end{aligned}$$

Firstly, we estimate $J_{1}$. We will show that the following estimate holds:

4.12 $$ \bigl\vert F^{\prime}(\xi) \bigr\vert \geq \frac{ \vert x \vert }{2},\quad \xi\in[0,\delta \rho]. $$

Now we divide the verification of (4.12) into two cases according to the value of *ξ*.

*Case* (I-a): $\xi\in[0,1)$. Since $m_{1}>1$ and $0< d<1$, we have

4.13 $$ d \bigl\vert \phi^{\prime}(\xi) \bigr\vert \leq C_{5} \,d \xi^{m_{1}-1}\leq C_{5}\leq \frac {M}{2}\leq\frac{ \vert x \vert }{2}. $$

By (4.13), if $\xi\in[0,1)$, we get

4.14 $$ \bigl\vert F^{\prime}(\xi) \bigr\vert \geq \vert x \vert -d \bigl\vert \phi^{\prime}(\xi) \bigr\vert \geq \frac{ \vert x \vert }{2}. $$

*Case* (I-b): $\xi\in[1,\delta\rho]$. Since $m_{2}>1$, we have

4.15 $$ d \bigl\vert \phi^{\prime}(\xi) \bigr\vert \leq C_{2} \,d \xi^{m_{2}-1}\leq C_{2}\,d \delta ^{m_{2}-1}\frac{ \vert x \vert }{d}\leq C_{2} \delta^{m_{2}-1} \vert x \vert \leq\frac{ \vert x \vert }{2}. $$

By (4.15), we get

4.16 $$ \bigl\vert F^{\prime}(\xi) \bigr\vert \geq \vert x \vert -d \bigl\vert \phi^{\prime}(\xi) \bigr\vert \geq \frac{ \vert x \vert }{2}. $$

Therefore (4.12) follows from (4.14) and (4.16). Since $\phi^{\prime}$ is monotonic on $\mathbb{R}^{+}$ by condition (H3) and $d>0$, it follows that $F^{\prime}$ is monotonic on $\xi\in I_{1}$. Thus, by (i) of Lemma 4.1 and estimate (4.12), (4.4), we have

4.17 $$ \vert J_{1} \vert \leq C\frac{1}{ \vert x \vert } \leq C \frac{1}{ \vert x \vert ^{\beta}}, $$

where we use $|x|\geq2$ and the fact $\frac{1}{2}\leq\beta\leq1$. Next we prove estimate $J_{3}$. Since $\xi\geq\lambda(\frac{|x|}{d})^{\frac{1}{m_{2}-1}}>1$ and $\lambda\geq(\frac{2}{C_{1}})^{\frac{1}{m_{2}-1}}$,

$$d \bigl\vert \phi^{\prime}(\xi) \bigr\vert \geq C_{1} \,d \xi^{m_{2}-1}\geq C_{1} \,d\lambda ^{m_{2}-1} \frac{ \vert x \vert }{d}\geq2 \vert x \vert , $$

it follows that

4.18 $$ \bigl\vert F^{\prime}(\xi) \bigr\vert \geq2 \vert x \vert - \vert x \vert = \vert x \vert ,\quad \xi\in[\lambda\rho ,\infty). $$

Thus, by (i) of Lemma 4.1 and estimate (4.18), (4.4), we have

4.19 $$ \vert J_{3} \vert \leq C\frac{1}{ \vert x \vert } \leq C \frac{1}{ \vert x \vert ^{\beta}}, $$

where we use $|x|\geq2$ and the fact $\frac{1}{2}\leq\beta\leq1$. Now, we give estimate $J_{2}$. Since $\xi\in I_{2}$, we have $|\xi|\geq1$. By (4.1), we obtain

4.20 $$ \bigl\vert F^{\prime\prime}(\xi) \bigr\vert \geq d \bigl\vert \phi^{\prime\prime}(\xi) \bigr\vert \geq C_{3}\,d \xi^{m_{2}-2} \geq C_{3}\,d \biggl(\frac{ \vert x \vert }{d} \biggr)^{\frac {m_{2}-2}{m_{2}-1}}. $$

We first prove that the following estimate holds:

4.21 $$ \max_{I_{2}} \vert \psi \vert + \int_{I_{2}} \bigl\vert \psi^{\prime} \bigr\vert \,d\xi \leq C \biggl(\frac{ \vert x \vert }{d} \biggr)^{-\frac{\alpha}{m_{2}-1}}. $$

In fact, since $\mu\in C_{0}^{\infty}(\mathbb{R})$ and $\frac {1}{2}\leq\alpha\leq\frac{m_{2}}{2}$, we get

4.22 $$ \max_{\xi\in A_{2}} \bigl\vert \psi(\xi) \bigr\vert \leq C(\delta\rho)^{-\alpha }=C\delta^{-\alpha}(\rho)^{-\alpha}= C \delta^{-\alpha} \biggl(\frac{ \vert x \vert }{d} \biggr)^{-\frac{\alpha}{m_{2}-1}}. $$

By (4.6), we have

4.23 $$\begin{aligned} \int_{A_{2}} \bigl\vert \psi^{\prime}(\xi) \bigr\vert \,d\xi &\leq\alpha \int_{A_{2}}\xi{\bigl(1+\xi^{2}\bigr)^{-\frac{\alpha}{2}-1}} \biggl\vert \mu \biggl(\frac{\xi}{N} \biggr) \biggr\vert \,d\xi+ \int_{A_{2}}{\bigl(1+\xi^{2}\bigr)^{-\frac{\alpha}{2}}} \frac{1}{N} \biggl\vert \mu^{\prime} \biggl(\frac{\xi}{N} \biggr) \biggr\vert \,d\xi \\ &=:L_{1}+L_{2}. \end{aligned}$$

Since $\mu\in C_{0}^{\infty}(\mathbb{R})$ and $\frac{1}{2}\leq \alpha\leq\frac{m_{2}}{2}$, we obtain

4.24 $$ L_{1}\leq C \int_{A_{2}}\xi{\bigl(1+\xi^{2}\bigr)^{-\frac{\alpha}{2}-1}} \,d\xi \leq C \int_{\delta\rho}^{\lambda\rho}\xi^{-\alpha-1}\,d\xi =C \biggl( \frac{ \vert x \vert }{d} \biggr)^{-\frac{\alpha}{m_{2}-1}} $$

and

4.25 $$ L_{2}\leq C (\delta\rho)^{-\alpha} \int_{A_{2}}\frac{1}{N} \biggl\vert \mu^{\prime} \biggl(\frac{\xi}{N} \biggr) \biggr\vert \,d\xi\leq C \biggl( \frac{ \vert x \vert }{d} \biggr)^{-\frac{\alpha}{m_{2}-1}}. $$

By (4.23), (4.24), and (4.25), we get

4.26 $$ \int_{ 0}^{\infty} \bigl\vert \psi^{\prime}(\xi) \bigr\vert \,d\xi\leq C \biggl(\frac { \vert x \vert }{d} \biggr)^{-\frac{\alpha}{m_{2}-1}}. $$

Therefore, (4.21) follows from (4.22) and (4.26). Thus, by (ii) of Lemma 4.1 and estimate (4.20), (4.21), we have

4.27 $$ \vert J_{2} \vert \leq d^{-\frac{1}{2}} \biggl( \frac{ \vert x \vert }{d} \biggr)^{-\frac {m_{2}-2}{2(m_{2}-1)}} \biggl(\frac{ \vert x \vert }{d} \biggr)^{-\frac{\alpha}{m_{2}-1}} =C\frac{d^{\frac{\alpha-\frac{1}{2}}{m_{2}-1}}}{ \vert x \vert ^{\frac{\alpha+\frac{m_{2}}{2}-1}{m_{2}-1}}}\leq C\frac{1}{ \vert x \vert ^{\beta}}. $$

Here in the last inequality we use the fact $\frac{\alpha-\frac{1}{2}}{m_{2}-1}\geq0$ and $0< d<1$. Therefore, for $|x|\geq M$, by estimates (4.11), (4.17), (4.19), and (4.27), it follows (4.3).

*Case* (II): $|x|< M$. Now we divide the verification of (4.3) into three cases according to the value of *α* for $|x|< M$.

*Case* (II-a): $\alpha>1$. Since $\mu\in C_{0}^{\infty}(\mathbb{R})$ and $\alpha>1$, we get

4.28 $$ \vert J \vert = \biggl\vert \int_{0}^{\infty} \frac{e^{i(d\phi( \vert \xi \vert )-x\xi )}}{(1+\xi^{2})^{\frac{\alpha}{2}}} \mu\biggl( \frac{\xi}{N}\biggr)\,d\xi \biggr\vert \leq C \int_{0}^{\infty} \frac {1}{{(1+\xi^{2})^{\frac{\alpha}{2}}}}\,d\xi\leq C. $$

Noting that $|x|< M$ and $\frac{1}{2}\leq\beta\leq1$, by (4.28), we have

$$\vert J \vert \leq C=C \vert x \vert ^{\beta}\frac{1}{ \vert x \vert ^{\beta}} \leq CM^{\beta}\frac {1}{ \vert x \vert ^{\beta}}=C\frac{1}{ \vert x \vert ^{\beta}}, $$

which follows (4.3).

*Case* (II-b): $\frac{1}{2}\leq\alpha<1$. By the mean value theorem, when $\frac{1}{2}\leq\alpha<1$, we have

4.29 $$ 0< \bigl(1+\xi^{2}\bigr)^{\frac{\alpha}{2}}- \xi^{\alpha} =\bigl(1+\xi^{2}\bigr)^{\frac{\alpha}{2}}-\bigl( \xi^{2}\bigr)^{\frac{\alpha}{2}} \leq\frac{\alpha}{2}\bigl( \xi^{2}\bigr)^{\frac{\alpha}{2}-1}\leq\xi ^{\alpha-2}. $$

By (4.29), we obtain

4.30 $$ \frac{1}{\xi^{\alpha}}-\frac{1}{(1+\xi^{2})^{\frac{\alpha}{2}}} =O \biggl(\frac{1}{\xi^{\alpha+2}} \biggr),\quad \xi\rightarrow\infty. $$

Noting that $\frac{1}{2}\leq\alpha<1$, by (4.30), we have

4.31 $$ \int_{0}^{\infty} \biggl\vert \frac{1}{\xi^{\alpha}}- \frac{1}{(1+\xi^{2})^{\frac{\alpha}{2}}} \biggr\vert \,d\xi \leq C $$

and

$$\begin{aligned} \vert J \vert = \biggl\vert \int_{0}^{\infty} \frac{e^{i(d\phi( \vert \xi \vert )-x\xi )}}{(1+\xi^{2})^{\frac{\alpha}{2}}} \mu\biggl( \frac{\xi}{N}\biggr)\,d\xi \biggr\vert \leq{}& \biggl\vert \int_{0}^{\infty}e^{i(d\phi( \vert \xi \vert )-x\xi)} \biggl( \frac{1}{(1+\xi^{2})^{\frac{\alpha}{2}}} -\frac{1}{\xi^{\alpha}} \biggr)\mu\biggl(\frac{\xi}{N}\biggr) \,d\xi \biggr\vert \\ &{} + \biggl\vert \int_{0}^{\infty}e^{i(d\phi( \vert \xi \vert )-x\xi)} \frac{1}{\xi^{\alpha}}\mu \biggl(\frac{\xi}{N}\biggr)\,d\xi \biggr\vert \\ ={}& :K_{1}+K_{2}. \end{aligned}$$

By (4.31), we have

4.32 $$ \vert K_{1} \vert \leq C=C \vert x \vert ^{\beta}\frac{1}{ \vert x \vert ^{\beta}}\leq CM^{\beta}\frac {1}{ \vert x \vert ^{\beta}}=C \frac{1}{ \vert x \vert ^{\beta}}. $$

By Lemma 1.3, we obtain

4.33 $$ \vert K_{2} \vert \leq C\frac{1}{ \vert x \vert ^{1-\alpha}}. $$

Noting that $\frac{1}{|x|}>\frac{1}{M}$, and the fact $\beta \geq1-\alpha$, it follows from $\frac{1}{2}\leq\alpha<1$, $\frac {1}{2}\leq\beta\leq1 $ that

4.34 $$ \vert x \vert ^{1-\alpha}= \vert x \vert ^{\beta} \vert x \vert ^{1-\alpha-\beta} = \vert x \vert ^{\beta} \biggl(\frac{1}{ \vert x \vert } \biggr)^{\beta-(1-\alpha)}\geq C \vert x \vert ^{\beta}. $$

Therefore, by (4.33) and (4.34), we have

4.35 $$ \vert K_{2} \vert \leq C\frac{1}{ \vert x \vert ^{\beta}}. $$

Hence, (4.3) holds from (4.32) and (4.35).

*Case* (II-c): $\alpha=1$. From the proof of Lemma 1.3, noting that $M\geq2$, we may get

4.36 $$ \vert J \vert \leq C \log\biggl(\frac{1}{ \vert x \vert }\biggr) \quad\text{if } 0< \vert x \vert \leq \frac{1}{2} $$

and

4.37 $$ \vert J \vert \leq C \quad\text{if } \frac{1}{2}< \vert x \vert < M. $$

By (4.36) and $\frac{1}{2}\leq\beta\leq1$, for $0<|x|\leq\frac{1}{2}$, we have

4.38 $$ \vert J \vert \leq C \log\biggl(\frac{1}{ \vert x \vert }\biggr)\leq C\frac{1}{ \vert x \vert ^{\beta}}. $$

By (4.37), for $\frac{1}{2}<|x|<M$, we have

4.39 $$ \vert J \vert \leq C=C \vert x \vert ^{\beta} \frac{1}{ \vert x \vert ^{\beta}}\leq CM^{\beta}\frac {1}{ \vert x \vert ^{\beta}}=C\frac{1}{ \vert x \vert ^{\beta}}. $$

Thus, for $\alpha=1$, $|x|< M$, by (4.38) and (4.39), we get

$$\vert J \vert \leq C\frac{1}{ \vert x \vert ^{\beta}}, $$

which is just estimate (4.3).

Summing up all the above estimates, we complete the proof of estimate (4.3) and finish the proof of Lemma 1.4. □

## Conclusion

In this paper, by linearizing the maximal operator and duality methods, and applying the results of Lemma 1.3 and Lemma 1.4, we obtain the maximal global $L^{q}$ inequalities (1.8) and (1.9) for multiparameter oscillatory integral $S_{t,\varPhi}$. These estimates are apparently good extensions to maximal global $L^{q}$ inequalities (1.6) and (1.7) for the multiparameter fractional Schrödinger equation in [32].
